# Phenotypic Changes and Oxidative Stress in THP-1 Macrophages in Response to Vanilloids Following Stimulation with Allergen Act d 1 and LPS

**DOI:** 10.3390/antiox14080949

**Published:** 2025-08-01

**Authors:** Milena Zlatanova, Jovana Grubač, Jovana Trbojević-Ivić, Marija Gavrović-Jankulović

**Affiliations:** 1 Department of Biochemistry, Faculty of Chemistry, University of Belgrade, 11000 Belgrade, Serbia; zlatanova@chem.bg.ac.rs; 2 Institute of Chemistry, Technology and Metallurgy, University of Belgrade, 11000 Belgrade, Serbia; jovana.grubac@ihtm.bg.ac.rs; 3 Innovative Centre, Faculty of Chemistry, Belgrade, Ltd., 11000 Belgrade, Serbia; jivic@chem.bg.ac.rs

**Keywords:** oxidative stress, surface markers, NF-κB, pro-inflammatory cytokines, actinidin, THP-1 macrophages, vanilloids

## Abstract

Activation of macrophages plays a key role in both inflammation and oxidative stress, key features of many chronic diseases. Pro-inflammatory M1-like macrophages, in particular, contribute to pro-oxidative environments and are a frequent focus of immunological research. This research examined the effects of kiwifruit allergen Act d 1, in comparison to LPS, on THP-1 macrophages in vitro differentiated under optimized conditions, both in the presence and in the absence of selected vanilloids. THP-1 monocyte differentiation was optimized by varying PMA exposure and resting time. Act d 1 induced M1-like phenotypic changes comparable to LPS, including upregulation of CD80, IL-1β and IL-6 secretion, gene expression of iNOS and NF-κB activation, in addition to increased reactive oxygen species (ROS) and catalase activity. Treatment with specific vanilloids mitigated these responses, primarily through reduced oxidative stress and NF-κB activation. Notably, vanillin (VN) was the most effective, also reducing CD80 expression and IL-1β levels. These results suggest that vanilloids can affect pro-inflammatory signaling and oxidative stress in THP-1 macrophages and highlight their potential to alter inflammatory conditions characterized by similar immune responses.

## 1. Introduction

Phenotypic studies on antigen-presenting cells (APCs) are pivotal to deciphering complex functional alterations in immune cells upon allergen stimulation [1,2,3]. Macrophages are tissue-resident phagocytes that serve as essential components of the innate immune system and are thereby invaluable to immunological studies [4,5]. Being highly plastic cells, macrophages change cell surface markers as a response to stimuli, switching their phenotype [6,7]. Two predominant macrophage phenotypes, M1 and M2, have been recognized and extensively studied. M1 phenotype is characterized by the release of pro-inflammatory cytokines (IL-1β, IL-6, IL-12, TNFα) and expression of surface markers CD80 (also known as B7-1, an inducible co-stimulatory molecule) and CD86 (B7-2) [6,7,8]. M2 phenotype is defined by the secretion of modulatory mediators (IL-10, IL-4, TGFβ) and expression of scavenger receptor CD163 and mannose receptor CD206 as surface markers [6,8,9]. Therefore, the specific activation profile of macrophages directly co-relates with the pro-inflammatory nature of the allergen and can also target new markers of sensitization [10].

Studies based on human donor-derived macrophages are hampered by genetic variability and difficulties in obtaining a sufficient number of cells [5]. THP-1 is a human monocytic cell line and represents an in vitro alternative to human primary macrophages, offering an unlimited number of cells and enabling the use of established reporter cell lines [11,12]. In vitro, macrophages can be distinguished from monocytes by their enlarged size and cytoplasmic content, consisting mainly of mitochondria and lysosomes. Therefore, prior to any treatment, THP-1 monocytes need to be differentiated to macrophages. Differentiation is typically initiated by phorbol-12-myristate-13-acetate (PMA), but conditions of PMA exposure vary between protocols, critically affecting cell morphology and size. The majority of the studies focus solely on cell surface markers expression and gene expression [4,13], and only some reflect whether the differentiated cells have the morphology of a macrophage [14]. Incomplete differentiation yields cells resembling monocytes more than macrophages, causing inconsistent response to stimuli and ambiguous results. Although it is not likely to define a standardized differentiation protocol, it is essential to recognize the importance of each differentiation step and optimize it accordingly.

Numerous convergent studies imply that enzymatic activity promotes pro-inflammatory activity of an allergen, extending it far beyond sensitization process. Actinidin (Act d 1, EC: 3.4.22.14) is a potent kiwifruit-specific allergen with cysteine protease activity and a complex mode of action [15]. The immunoglobulin E (IgE) reactivity of both native and thermally inactivated Act d 1, observed in vivo as well as in vitro, indicates that actinidin not only harbors surface allergenic epitopes but also contains internal hidden epitopes that are unmasked upon thermal treatments [15]. Consequently, its allergenicity cannot be reduced by cooking, the most common method of food processing [15]. A distinctive characteristic of protease allergens is their capability to bypass host tolerance. Due to its immanent protease activity, Act d 1 disrupts tight junctions between intestinal epithelial cells, facilitating allergen translocation across the gut epithelial barrier followed by uptake by APC. Act d 1 and other protease allergens mediate epithelial injury and activate protease-activated receptors (PARs), collectively eliciting robust inflammatory responses [10,16,17,18,19,20].

Inflammation is the basis of a wide range of diseases, including allergies, cancer, neurodegenerative, autoimmune and cardiovascular diseases. Inflammatory response can be activated by various mechanisms that result in activation of pattern recognition receptors (PRR) [21]. Subsequent stimulation of inflammatory and signaling factors (NF-κB, JNK, JAK/STAT) results in amplification of inflammation via production of specific cytokines (IL-1β, IL-6, TNFα) and activation of immune cells residing in the affected tissues [21,22,23]. Hence, the interest of the scientific community in different food bioactive components and their role in inflammation and alleviating oxidative stress is steadily increasing [24,25,26,27]. The rapid development of functional foods and nutraceuticals research in recent decades provided an evidence-based link between food and its specific health benefits. Vanilloids are a complex class of vanillyl alcohol-derived nutraceuticals and major bioactive compounds of various medicinal plants and food sources, including *Gastrodia elata*, *Phalenopsis*, *Vanilla planifolia*, *Capsicum anuum*, etc. [28,29,30]. Their anti-microbial and anti-inflammatory effects are reported in numerous studies, even on strong pro-inflammatory stimuli such as lipopolysaccharide (LPS) [30,31,32,33,34,35,36,37,38,39]. In the context of food allergens and food proteases, it is beneficial to characterize the extent of the anti-inflammatory effect provided by other molecules commonly found in food, such as vanilloids, and whether this effect can be observed on immune cells and in small doses.

The goal of this study was to establish a reproducible as well as reliable immune cell system for evaluation of phenotypic and genotypic alterations in macrophages under the impact of food biomolecules. Act d 1 was selected as a prototype of a potent food allergen displaying proteolytic activity, while bioactive compounds of vanilloid family were selected as its counterbalance. Optimal conditions for THP-1 differentiation into macrophages were examined to establish a stable and uniform cell culture, followed by in-depth analysis of oxidative stress markers, NF-κB signaling and phenotypic changes in our macrophage model upon Act d 1 and vanilloid treatment.

## 2. Materials and Methods

### 2.1. Allergen Preparation and Purification

Act d 1 was isolated and purified from fresh kiwifruit (*Actinidia deliciosa*, Hayward cv) purchased from a local store. The purification procedure involved two consecutive ion-exchange chromatography steps, as previously described [19,40]. From 250 g of fresh fruit, a total of 110 mg of Act d 1 was obtained. The purified protein was concentrated to a concentration of 1 mg/mL, which was estimated using BCA Protein Assay Kit (Thermo Scientific, Waltham, MA, USA). After concentration, the allergen was lyophilized and stored at −20 °C. The purity of the protein was estimated to be >95% based on SDS-PAGE.

### 2.2. Preparation of Vanilloids

Vanillyl alcohol (VA), vanillyl laurate (VL) and vanillin (VN) (≥98% purity, food grade) were purchased from Sigma-Aldrich, St. Louis, MO, USA. The compounds were dissolved in 0.05% dimethyl sulfoxide (DMSO) and subsequently used for treatment purposes in the final concentrations of 5 and 25 µM. These particular concentrations were selected based on literature reports as the minimal concentrations of structural analogues that exhibit physiological effects [41,42].

### 2.3. Caseinolytic Activity

Native Act d 1 represents a mixture of zymogen and an active enzyme [15]. Therefore, prior to enzymatic assay, Act d 1 was activated in 20 mM potassium phosphate buffer, containing 20 mM L-cysteine and 2 mM EDTA (1:1; *v*/*v*), for 1 h at 37 °C.

Enzymatic activity of purified Act d 1 was verified in caseinolytic assay [43]. In brief, casein (Sigma-Aldrich, St. Louis, MO, USA) was prepared as a 2% solution in 100 mM potassium phosphate buffer, pH 7.6, and were added to the assay mixture in a volume of 0.4 mL, together with 40 µL of 250 mM L-cysteine, 40 µL of 250 mM EDTA, 40 µL of 250 mM NaOH, 120 µL of 1 M KH_2_PO_4_, pH 7.0 and 80 µL of distilled water. After preparation of the reaction mixture, 80 µL of Act d 1 solution were added, and the mixture was incubated for 1 h at 37 °C. The enzymatic reaction was terminated by the addition of 1.2 mL of 10% trichloroacetic acid (TCA), after which the solution was incubated for an additional hour at 37 °C. Following centrifugation (12,000× *g*, Centrifuge MiniSpin, Eppendorf, Hamburg, Germany) at room temperature for 15 min, absorbance at 280 nm was measured in the supernatant against a blank. The blank was prepared in the same manner as an assay mixture, except that TCA was added before Act d 1. One unit of caseinolytic activity was defined as the quantity of enzyme that gives 0.001 A_280_ per minute at 37 °C.

To examine whether vanilloids used in the study affect the enzymatic activity, they were incubated with Act d 1 in the reaction mixture, in a volume of 80 µL, replacing water in the control sample, in different molar ratios (1/0.3, 1, 2, 6 and 10, Act d 1/vanilloid). The activity was then compared to the activity of untreated Act d 1 and was expressed as a percentage of initial Act d 1 activity.

### 2.4. Cultivation of THP-1 Monocytes

THP-1 human leukaemia monocytes (ATCC, Manassas, VA, USA), were seeded and grown in culture in RPMI 1640 (Gibco, Thermo Fisher Scientific, Waltham, MA, USA), in addition to 10% heat-inactivated fetal bovine serum (FBS, Biowest, Nuaille, France), penicillin (100 IU)/streptomycin (100 µg/mL), 0.05 mM 2-mercaptoethanol and 200 mM L-glutamine (Sigma-Aldrich, St. Louis, MO, USA). Cells were cultured in an atmosphere of 5% CO_2_ at 37 °C.

### 2.5. Optimization of THP-1 Monocytes Differentiation Time with PMA

In order to optimize the differentiation conditions and analyze the effect of various incubation times on cell morphology, THP-1 monocytes were differentiated into macrophages in 24-well plates at a cell density of 4.8 × 10^5^ cells/well in RPMI 1640 medium containing 100 ng/mL phorbol-12-myristate-13-acetate (PMA, Sigma-Aldrich, St. Louis, MO, USA). The time that the cells spent in medium containing PMA was varied, using 24, 48 or 72 h. The cells were then switched to rest over different time periods (24, 48 or 72 h) in a complete RPMI 1640 medium without PMA. After fixing the cells with 4% paraformaldehyde for 15 min, washing with PBS solution pH 7.4 and staining the cell nuclei with 4′,6-diamidino-2-phenylindole (DAPI, Thermo Fisher Scientific, Waltham, MA, USA), the size of the differentiated cells and nuclei was examined using a fluorescence microscope (Opto-Edu, Beijing, China) equipped with a 10× and 40× magnification objective and a digital microscopic camera connected to Image View 3.7 software for image capture and analysis. DAPI-stained nuclei were imaged using a fluorescence filter with a 440–460 nm emission range. Each cell was analysed morphologically by separately marking and outlining the cell and nuclei areas in the imaging software. The cell area was determined under visible light, while the nuclear area was measured using the DAPI filter, with both areas expressed in µm^2^. The software-calculated cell and nuclear areas were extracted and used for calculation of the nuclei-to-cell area ratio. The final results were visualized and analyzed using GraphPad Prism 8.0.

### 2.6. Cell Treatment

Before treatments, THP-1 monocytes were differentiated into macrophages in 48-well plates (2.4 × 10^5^ cells/well) or in 96-well plates (8 × 10^4^ cells/well) in RPMI 1640 medium with 100 ng/mL PMA. After that, the cells were left to rest over 72 h in a complete RPMI 1640 medium without PMA. Before the treatment of differentiated macrophages, Act d 1 was activated in the medium, which contains 0.8 mM cysteine, for 1 h at 37 °C. Act d 1 was used in a concentration of 16.62 μM (1 mg/mL) for all cell treatments, as previously optimized on THP-1 differentiated macrophages [17]. To analyze the effects of non-active Act d 1, the protein was pre-incubated at 37 °C for 1 h with 10 µM E64 cysteine protease inhibitor (N-[N-(L-3-trans-carboxyoxiran-2-carbonyl)-L-leucyl]-agmatine, Sigma-Aldrich, St. Louis, MO, USA). LPS concentration of 0.1 µg/mL is a concentration often used in studies for analyzing responses of immune cells [44,45,46]. In the treatments, vanilloids were added 1 h prior to LPS or Act d 1 treatment at two different concentrations (5 and 25 μM). Act d 1 or LPS was added after 1 h, following continuation of the treatment for 24 h. LPS from *Escherichia Coli* O127:B8 was purchased from Sigma-Aldrich, St. Louis, MO, USA. Following this time period, cell supernatants were collected if necessary, and the cells were used for the appropriate assay.

### 2.7. Measurement of NO Levels

An indicator of nitric oxide synthase (NOS) activity, NO, was quantified in cell supernatants collected after treatment, following the Griess method [47]. In brief, equivalent volumes of 0.1% naphthyl-ethylenediamine dihydrochloride and 1% sulfanilamide (Sigma-Aldrich, St. Louis, MO, USA) in 5% H_3_PO_4_ were combined and mixed with the cell supernatant (1/1, *v*/*v*). Following incubation at room temperature for 30 min, the absorbance was analyzed at 548 nm, using a microplate reader (BioTek Synergy LX Multimode Reader, Agilent, CA, USA).

### 2.8. Measurement of Reactive Oxygen Species (ROS)

Production of reactive oxygen species (ROS) was measured using a cell-permeable probe, 2′,7′-dichlorodihydrofluorescein diacetate (DCFH-DA, Cayman Chemical, MI, USA). DCFH-DA is de-esterified intracellularly and oxidized by ROS to form fluorescent 2′,7′-dichlorofluorescein. Macrophages were differentiated and treated in 96-well plates as previously described, and after 24 h of treatment, the medium was replaced with RPMI 1640 phenol-free media, and DCFH-DA solution was added to a final concentration of 50 µM and incubated for 30 min. ROS levels were further quantified by spectrofluorometric analysis on excitation and emission wavelengths of λ 488 nm and λ 525 nm, respectively, using a microplate reader (BioTek Synergy LX Multimode Reader, Agilent, CA, USA).

### 2.9. Catalase Activity

THP-1 differentiated cells were pre-treated with vanilloids (25 µM) before LPS or Act d 1 treatment and lysed after removing the cell supernatants, using 50 mM Tris buffer, 0.25 M sucrose, 1 mM EDTA, pH 7.4. After centrifugation at 12,000× *g*, supernatants were used for further analysis. BCA Protein Assay Kit (Thermo Scientific, Waltham, MA, USA) was used to determine the protein concentration in the samples following the manufacturer’s instructions. Catalase activity was followed after mixing the samples with 10 mM H_2_O_2_ in phosphate buffer pH 7 in a 2:1 ratio (*v*/*v*) and following the change of absorbance at 240 nm over 5 min using a microplate reader (BioTek Synergy LX Multimode Reader, Agilent, CA, USA). Decrease in absorbance of 0.1 per minute at 25 °C on pH 7 was defined as one unit of catalase activity. Enzyme activity was expressed as units (U) per mg of protein in each analyzed sample.

### 2.10. Measurement of GSH Levels

Levels of reduced glutathione (GSH) in THP-1 differentiated cells were analyzed using the Tietz method. Samples and standards were mixed with 3 mM NaHPO_4_ buffer in a 1 to 8 ratio (*v*/*v*), after which 1 volume of the 0.04% DTNB (5,5-dithiobis 2-nitrobenzoic acid, Sigma-Aldrich, St. Louis, MO, USA) substrate solution in 0.1% Na-citrate was added. The mixture was incubated for 10 min at room temperature, and the absorbance at 412 nm was measured using a microplate reader (BioTek Synergy LX Multimode Reader, Agilent, CA, USA). Reduced glutathione (AppliChem GmbH, Darmstadt, Germany) was used to prepare standard solutions for the construction of a standard curve in the range 1–100 µM.

### 2.11. Gene Expression

THP-1 differentiated macrophages were pre-treated with vanilloids before treatment with LPS or Act d 1 for 24 h. After the treatment, total RNA was isolated using TRI Reagent^®^ (Sigma-Aldrich, St. Louis, MO, USA) and was used as a starting template in quantitative PCR reaction. The reaction mixture was prepared using KAPA SYBR^®^ FAST One-Step qRT-PCR Master Mix kit (Merck, Darmstadt, Germany), adequate forward and reverse primers in the concentration of 10 µM each and 200 ng of isolated RNA following manufacturers instructions. The specific primers for both human inducible nitric oxide synthase (iNOS) and housekeeping gene GAPDH used for normalization were derived from GenBank NCBI (National Center for Biotechnology Information) and purchased from Invitrogen (San Diego, CA, USA) and are shown in Table 1 with their Tm values. The parameters of the PCR cycle were designed according to the manufacturers recommendations and are shown in Table 2. Relative gene expression was calculated using the 2^(−∆∆ct)^ calculation method, and expression levels were normalized to untreated control cell groups.

### 2.12. Flow Cytometric Analysis of Surface Markers

The expression of cell surface markers was measured by flow cytometry. THP-1 monocytes were cultured in T-75 flasks in a complete medium and were differentiated into macrophages as previously described. The adherent macrophages were detached from the plate surface using accutase (Biowest, Nuaille, France) and stained with appropriate anti-human CD antibodies (5 µL of commercial antibody per million cells) for 20 min at 4 °C, according to the manufacturer’s recommendation. All antibodies (anti-human CD86 and anti-human CD163 FITC-labeled antibodies and anti-human CD80 and anti-human CD206 PE-labeled antibodies) were supplied from BioLegend, San Diego, CA, USA. Cells were analyzed by flow cytometry using FACS Calibur (BD Biosciences, San Jose, CA, USA) equipped with a blue solid-state 200-mW laser (488 nm used for excitation) and appropriate detection filters. Raw data were analyzed using BD CellQuestPro software version 5.1 (BD Biosciences, San Jose, CA, USA).

### 2.13. ELISA Detection of Cytokines

Sandwich ELISA was employed for the detection and quantification of IL-1β and IL-6 in cell supernatants, according to the manufacturer’s instructions (Human IL-1β DuoSet ELISA and Human IL-6 DuoSet ELISA, R&D Systems, Minneapolis, MN, USA). Following overnight incubation of the capture antibodies on the plate in PBS pH = 7.4 at RT, wells were blocked with 1% BSA in PBS for 1 h at RT. Samples and standards were then added and incubated for 2 h at RT. Detection antibodies were incubated for 1 h at RT, after which streptavidin labeled with horseradish peroxidase (HRP) was added for 30 min. In between each of the mentioned steps, plates were washed 4 times with PBS pH 7.4 containing 0.05% Tween-20. TMB substrate (Biolegend, San Diego, CA, USA) was used for detection, and 2N H_2_SO_4_ was used for stopping the reaction. Absorbance was measured at 450 nm using a microplate reader (BioTek Synergy LX Multimode Reader, Agilent, CA, USA).

### 2.14. Luciferase Reporter Assay with NF-κB Response Element

Monocytes (1 × 10^5^) were seeded and differentiated to macrophages in 96-well plates as previously described and then transfected with NanoLuc^®^ Reporter Vector with NF-κB Response Element (Promega, Madison, WI, USA) using FuGENE^®^ HD Transfection Reagent (Promega, Madison, WI, USA), according to the manufacturer’s instructions, by using 50 µg of plasmid DNA per well and a 4/1 ratio of transfection reagent/plasmid DNA (*v*/*v*). After 24 h of transfection, the media was changed, and transfected cells were treated as previously described. After treatment for 24 h, luciferase activities in each well were determined by addition of equal volume as the supernatant in each well of Nano-Glo^®^ Luciferase Assay Reagent (Promega, Madison, WI, USA). After incubation for 10 min at room temperature, relative luminosity units (RLU) were measured using a microplate reader (BioTek Synergy LX Multimode Reader, Agilent, CA, USA).

### 2.15. Statistical Analysis

In the graphs included in this paper, the results are presented as mean ± SEM. Abbreviations to indicate *p*-values are as follows: * *p*-value < 0.05, ** *p*-value < 0.01, *** *p*-value < 0.001 and **** *p*-value < 0.0001. Visualization and subsequent statistical analysis were conducted in GraphPad Prism version 8.0.1 (GraphPad, La Jolla, CA, USA). For the analysis of statistical significance between groups, one-way analysis of variance (ANOVA) was used with an applied *p*-value threshold of 0.05 with a 95% confidence interval to determine statistical significance. All measurements were performed in technical duplicates and repeated at least three times, and results are shown from three independent experiments.

## 3. Results

### 3.1. Optimization of THP-1 Differentiation

**Figure 1:**
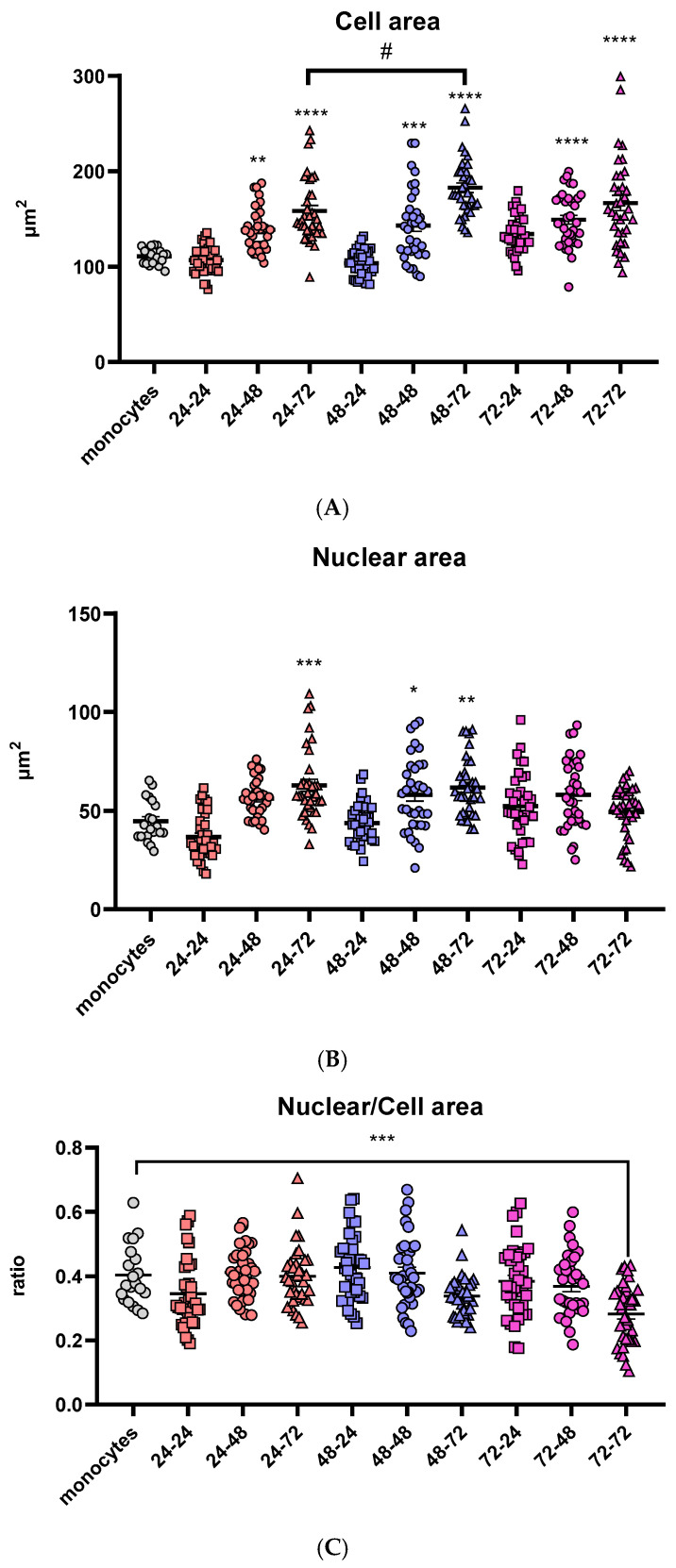
Comparison of morphologic characteristics of differentiated macrophages. Cell (**A**) and nuclear (**B**) areas are shown in µm^2^ and the ratios of nuclear and cell area (**C**) for all tested differentiation conditions. Results are presented in a scatter plot as individual values as well as mean ± SEM, n = 3 (at least 15 values per independent experiment); */# *p*-value < 0.05, ** *p*-value < 0.01, *** *p*-value < 0.001, and **** *p*-value < 0.0001, * compared to monocytes.

In order to visualize variations in the morphology of the cells and differentiation of THP-1 monocytes to macrophages, different incubation times in medium both with and without PMA were used. A total of nine different combinations were tested together with undifferentiated THP-1 monocytes as shown in Table 3. Morphological differences in size were observed and are in direct correlation with the incubation time in the medium without PMA after its removal. Prolongation of the “resting time” in PMA-free media above 48 h led to a substantial rise in cell area, regardless of the differentiation time in the PMA medium. The highest cell area was reached after 72 h of the resting period (Figure 1A). In contrast, cells that were differentiated for 24 h in PMA-free medium had no significant change in cell area compared to undifferentiated monocytes.

The nuclear area follows the same trend of time-dependent increase in cell area in PMA-free medium: in the groups that were incubated with PMA-free medium for 24 h, the nuclei area stayed similar to the monocytes. In the groups treated with PMA over 24 and 48 h, the nuclear area increased with the prolongation of the resting period. However, one difference was noticed in the groups treated with PMA for 72 h: there were no significant changes in nuclear area with the change of the resting period (Figure 1B).

Additionally, to visualize the change in nuclear and cell area and their correlation, the nuclear-to-cell area ratio was calculated for all nine differentiation conditions as well as the monocyte control group. The only statistically significant difference was noticed after 72 h in PMA, followed by a 72-h resting period (Figure 1C). Cell size of eukaryotic cells is strongly correlated with the nuclear size [48], indicating that there is an undesired change in cell morphology at this time point, possibly due to the long total time the cells had spent in a culture (6 days), or more likely the long exposure time in the presence of PMA (3 days), which can cause metabolic stress and undesired changes in expression of different genes.

**Figure 2:**
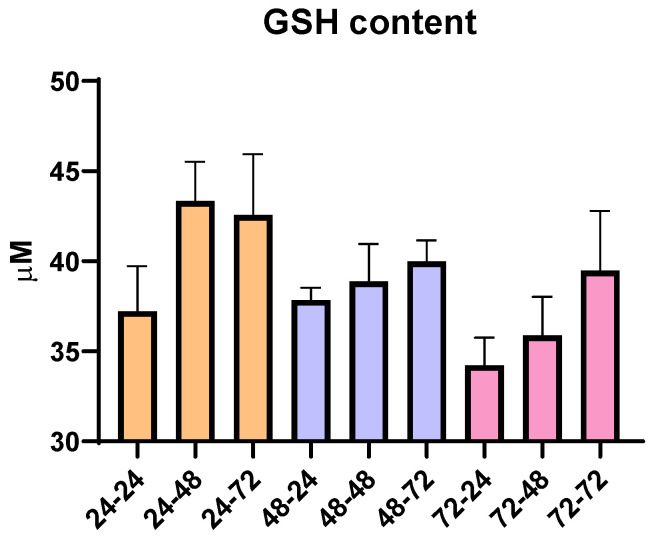
Concentration of reduced glutathione (GSH) in THP-1 macrophages for all tested differentiation conditions. Results are presented as mean ± SEM, n = 3; ns—non-significant.

To analyze whether the total time in cell culture as time spent in PMA and PMA-free media affected oxidative stress, an additional analysis of the total content of reduced glutathione (GSH) was conducted. Since GSH has the role of an antioxidant and can be an indicator of oxidative stress in the cells, according to our results, we did not observe any statistically significant changes over the nine different time periods used for THP-1 differentiation. However, though not statistically significant, there are changes that are in correlation with the pro-inflammatory ability of PMA and its ability to moderately increase oxidative state, since we observe that the prolongation of the resting time in media without PMA slightly increases GSH content (Figure 2). Overall, the concentration of GSH remained within the range of 30–45 µM, suggesting limited impact of PMA, resting time and overall time spent in culture, as well as the ability of cells to recover from potential PMA-induced pro-oxidative effects.

Based on the presented results, the optimal differentiation was achieved after 48 h in medium with PMA, followed by 72 h incubation in PMA-free medium. The obtained cells have a larger cell and nuclear area, compared to monocytes, an important characteristic of in vitro differentiated macrophages, stable nuclear/cell ratio similar to monocytes, and stable levels of GSH, indicating normal metabolic growth. Therefore, all subsequent experiments and treatments were carried out under these differentiation conditions.

### 3.2. Phenotypic Changes in THP-1 Macrophages After Act d 1 Stimulation and Vanilloid Treatment

**Figure 3:**
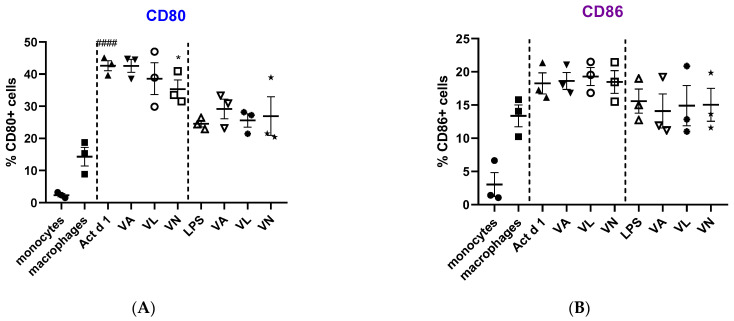
Expression of M1 surface markers, CD80 (**A**) and CD86 (**B**), on macrophages after pre-treatment with vanilloids followed by Act d 1 and LPS stimulation. Results are presented as % of positive cells, mean ± SEM, n = 3; * *p*-value < 0.05, #### *p*-value < 0.0001, * compared to Act d 1/LPS, # compared to untreated macrophages. VA—vanillyl alcohol, VL—vanillyl laurate, VN—vanillin.

**Figure 4:**
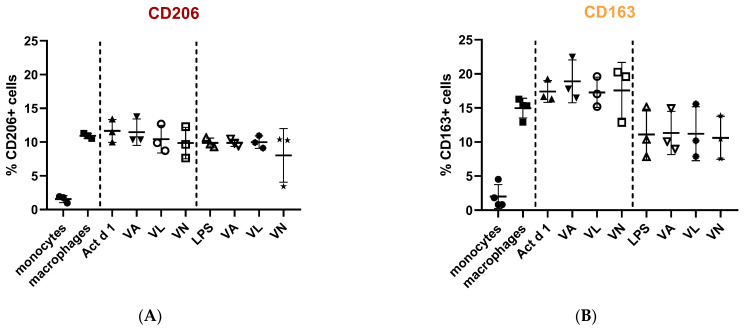
Expression of M2 surface markers, CD206 (**A**) and CD163 (**B**), on macrophages after pre-treatment with vanilloids followed by Act d 1 and LPS stimulation. Results are presented as % of positive cells, mean ± SEM, n = 3; VA—vanillyl alcohol, VL—vanillyl laurate, VN—vanillin.

Macrophage polarization and phenotypic changes are crucial for the events following the immune response to stimuli. In order to analyze potential changes in the phenotype on THP-1 cells after stimulation with Act d 1, alongside LPS stimulation, we used flow cytometry to test M1 surface markers CD80 and CD86, commonly increased in response to pro-inflammatory stimuli, and M2 surface markers, CD206 and CD163, increased in alternatively activated macrophages. Act d 1 increased CD80 expression in more than 40% of the macrophages, having a higher impact than LPS treatment (Figure 3A). The rest of the analyzed markers were not elevated, neither following Act d 1 nor LPS stimulation (Figure 3B and Figure 4A,B).

**Figure 5:**
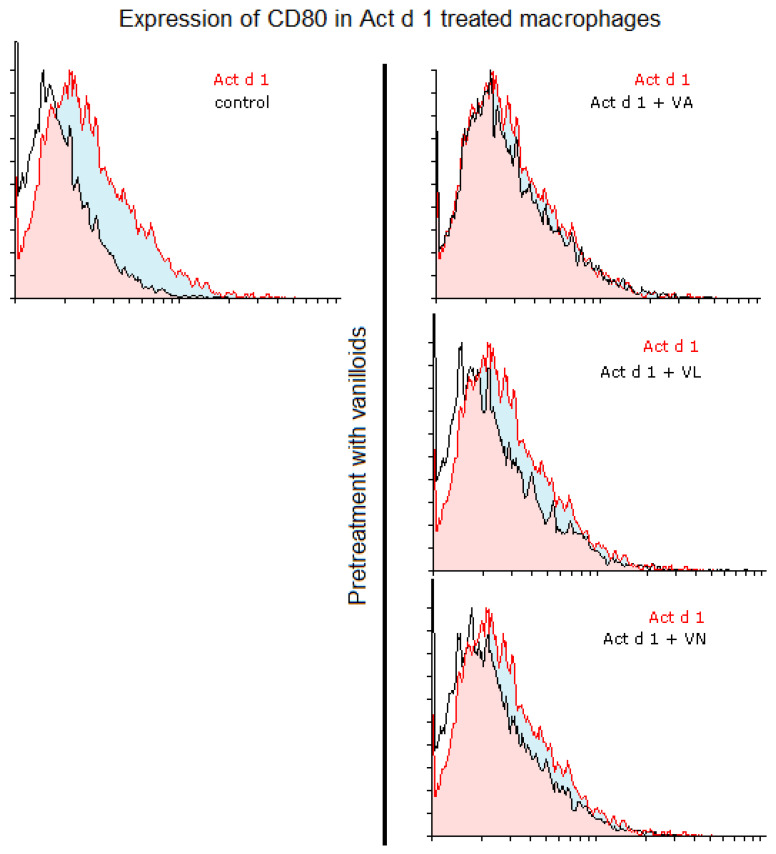
CD80 expression in Act d 1 treated macrophages. Histogram overlaps showing CD80 expression of Act d 1 stimulated THP-1 macrophages compared to control (**left**), as well as macrophages pre-treated with vanilloids (**right**). Representative experiments are shown. VA—vanillyl alcohol, VL—vanillyl laurate, VN—vanillin.

Additionally, we analyzed whether vanilloids will also have an impact on macrophage polarization. Indeed, although VL and VN both have lower values than Act d 1-treated samples, VN reduced CD80 expression after Act d 1 stimulation in THP-1 macrophages with statistical significance, though not entirely to control levels (Figure 3A and Figure 5), indicating its limited impact on macrophage polarization and in reducing the observed pro-inflammatory effect of Act d 1.

### 3.3. Vanilloids Have No Impact on the Protease Activity of Act d 1

**Figure 6:**
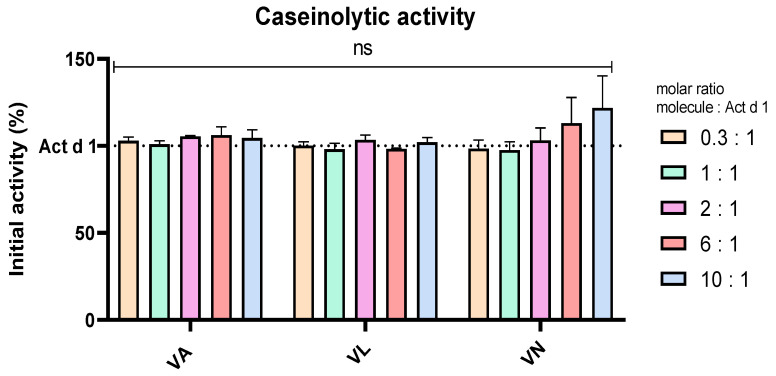
Caseinolytic activity of Act d 1 with vanilloids in different molar ratios. Initial activity of untreated Act d 1 is regarded as 100%. Results are presented as mean ± SEM, n = 3; ns—non-significant, compared to initial Act d 1 activity. VA—vanillyl alcohol, VL—vanillyl laurate, VN—vanillin.

Casein was used as a substrate in an assay to analyse the effect of vanilloids on the enzymatic activity of Act d 1, which is the proposed pro-inflammatory mechanism of this allergen, supported by the lack of inflammation upon enzyme inhibition with a specific cysteine protease inhibitor [18,40,49]. To exclude the possibility of Act d 1 inhibition by vanilloids, Act d 1 was incubated in the presence and absence of these molecules with casein as a substrate. Different molar ratios of the molecules were used, Act d 1/tested molecule 1/X, with X being 0.3, 1, 2, 6 or 10. These ratios were chosen as they cover the tested concentrations of the molecules, and they could portray if there is a noticeable inhibition of the enzyme in the treatment conditions used in this paper. There was no significant change in the enzymatic activity with any of the tested concentrations and molecules, compared to Act d 1 alone (Figure 6), implying that anti-inflammatory, antioxidant and potential immunomodulatory effects of vanilloids are not related to inhibition of Act d 1.

### 3.4. Markers of Oxidative Stress Decrease After Treatment with Vanilloids

**Figure 7:**
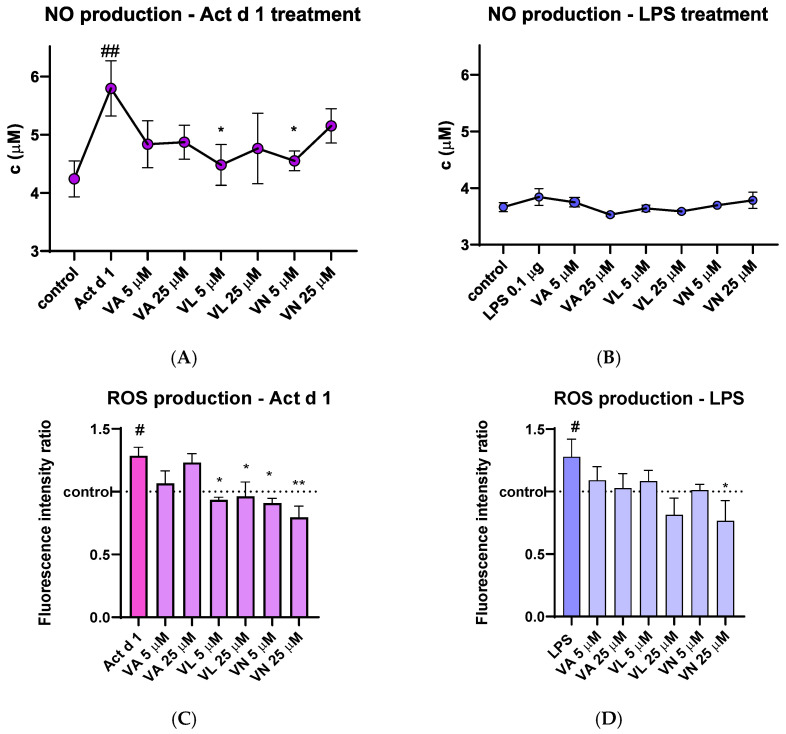
Effects of vanilloids on oxidative stress markers. NO production in THP-1 macrophages after pre-treatment with vanilloids and stimulation with Act d 1 (**A**) and LPS (**B**). Production of ROS in THP-1 cells after pre-treatment with vanilloids and stimulation with Act d 1 (**C**) and LPS (**D**). Results are presented as mean ± SEM, n = 3; #/* *p*-value < 0.05, ##/** *p*-value < 0.01, * compared to Act d 1/LPS, # compared to control. VA—vanillyl alcohol, VL—vanillyl laurate, VN—vanillin.

After treatment of THP-1 macrophages, cell supernatants were used to analyze the concentration of the NO metabolite. THP-1 macrophages treated with Act d 1 showed a noticeable increase in NO production, which was statistically lowered after pre-treatment with 5 µM VL and 5 µM VN (Figure 7A). LPS (0.1 µg/mL) did not induce a statistically significant increase in NO levels (Figure 7B), presumably due to its low concentration or the nature of the method used for NO quantification.

The amount of intracellular ROS was analyzed by spectrofluorimetric detection using DCFH-DA probe. Upon stimulation with LPS and Act d 1, an increase in fluorescence was detected, indicating the mild pro-oxidative effect of these molecules (Figure 7C,D). In both cases, pre-treatment with 25 µM VN reduced the fluorescence intensity to the levels of the control group of untreated THP-1 macrophages, indicating its antioxidative properties. Furthermore, in the Act d 1 treated group, VL and VN in both concentrations significantly decreased ROS production (Figure 7C).

**Figure 8:**
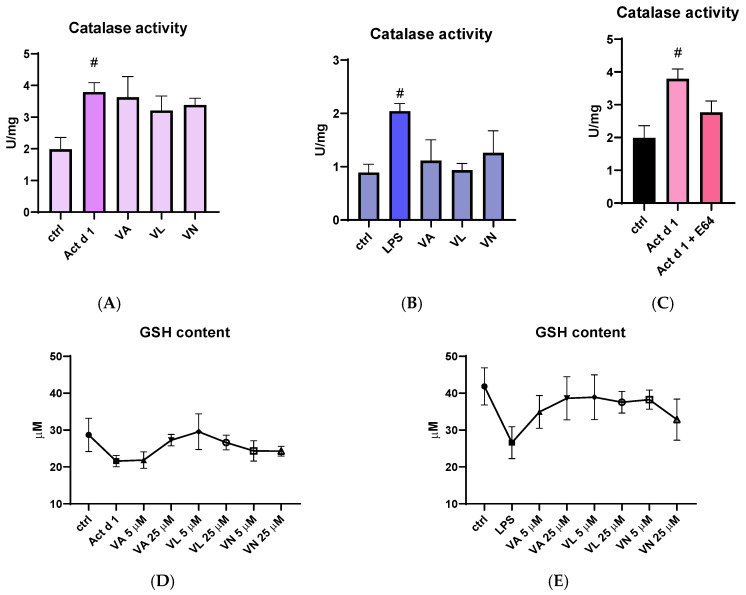
Effects of vanilloids on oxidative stress markers. Catalase activity in THP-1 macrophages after pre-treatment with vanilloids and stimulation with Act d 1 (**A**) and LPS (0.1 µg/mL) (**B**). Catalase activity after treatment with active and E64-inhibited Act d 1 (**C**). GSH content in THP-1 macrophages after pre-treatment with vanilloids and stimulation with Act d 1 (**D**) and LPS (0.1 µg/mL) (**E**). Results are presented as mean ± SEM, n = 3; # *p*-values < 0.05, compared to control. VA—vanillyl alcohol, VL—vanillyl laurate, VN—vanillin.

Catalase activity was measured in total cell lysates following appropriate treatments. Both Act d 1 and LPS increased catalase activity in THP-1 cells, suggesting higher oxidative stress. Although the higher concentrations of vanilloids used for pre-treatment (25 µM) did not significantly alter catalase activity after Act d 1 treatment (Figure 8A), they moderately reduced catalase activity following LPS treatment (Figure 8B). Importantly, the E64-inhibited Act d 1 did not induce a statistically significant increase in catalase activity like its active counterpart (Figure 8C). In addition, total reduced glutathione (GSH) levels were also analyzed as another indicator of oxidative stress, due to its antioxidant role in the cell. Both Act d 1 and LPS induced a moderate decrease in GSH concentration; however, the changes were not statistically significant, possibly due to the limited sensitivity of the assay used. Treatment with the vanilloids led to an increase in GSH levels, though these changes were also not statistically significant (Figure 8D,E).

### 3.5. Vanilloids Alter Production of Pro-Inflammatory Cytokines

**Figure 9:**
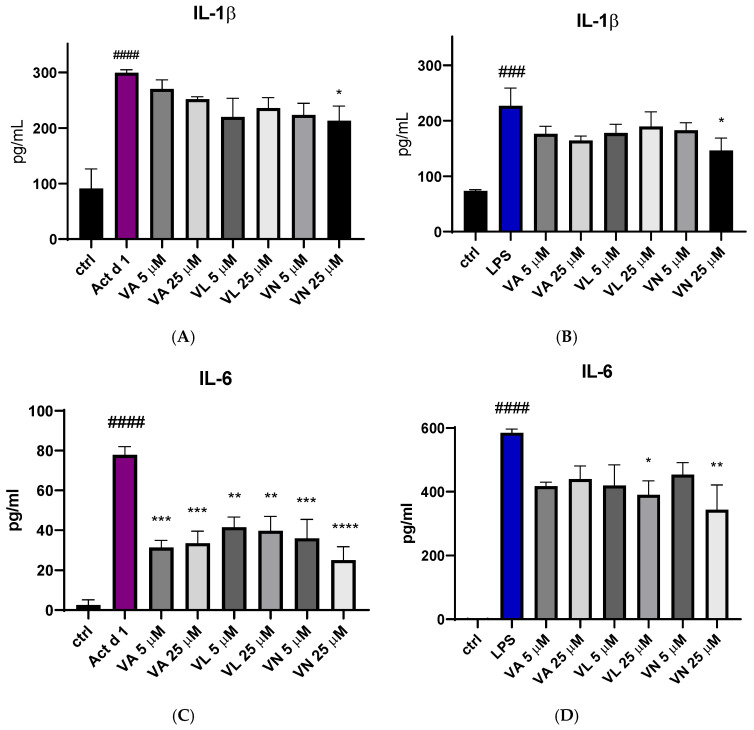
Secretion of proinflammatory cytokine IL-1β after stimulation with Act d 1 (**A**) and LPS (**B**) and IL-6 after stimulation with Act d 1 (**C**) and LPS (**D**) in THP-1 macrophages. Results are presented as mean ± SEM, n = 3; * *p*-value < 0.05, ** *p*-value < 0.01, ***/### *p*-value < 0.001, and ####/**** *p*-value < 0.0001, * compared to Act d 1/LPS, # compared to control. VA—vanillyl alcohol, VL—vanillyl laurate, VN—vanillin.

The effect of vanilloid pre-treatment on the production of pro-inflammatory cytokines IL-1β and IL-6 was assessed using ELISA. After treatment with Act d 1 and LPS, there was an increase in IL-1β secretion in the cell supernatants (Figure 9A,B) and IL-6 (Figure 9C,D). Reduced IL-1β production was noticed after pre-treatment with 25 µM VN in both LPS and Act d 1-induced inflammation (Figure 9A,B). All tested vanilloids led to decreased production of IL-6 following Act d 1 treatment (Figure 9C), while only VL and VN in the higher concentration used led to reduced IL-6 production following LPS treatment (Figure 9D). This difference could be a consequence of the initial increase in IL-6 production by each of the stimulants, with LPS increasing IL-6 close to 600 pg/mL, while Act d 1 increased IL-6 to around 80 pg/mL, highlighting the ability of vanilloids to significantly decrease but not completely diminish pro-inflammatory cytokine production.

**Figure 10:**
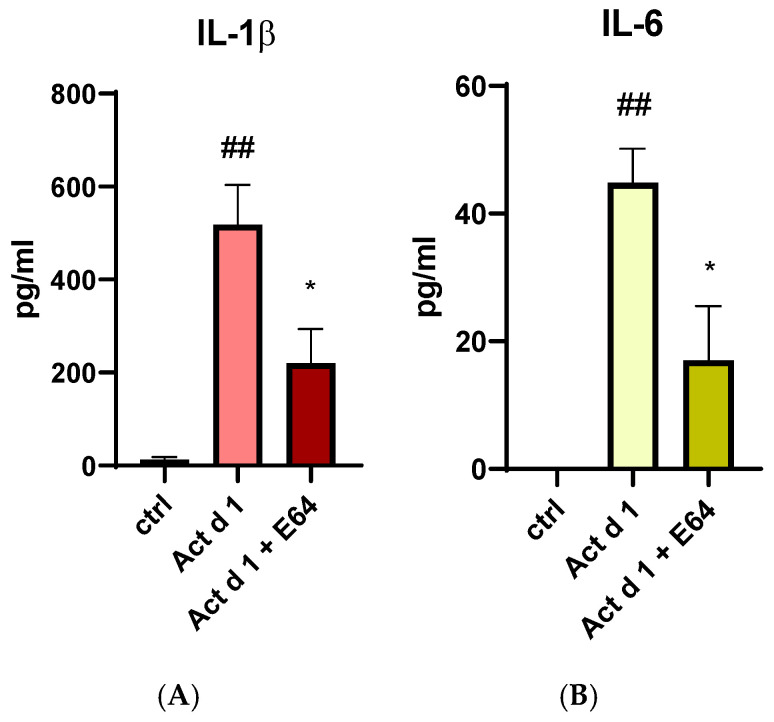
Secretion of proinflammatory cytokine IL-1β (**A**) and IL-6 (**B**) after stimulation with active Act d 1 and Act d 1 pre-incubated with cysteine protease inhibitor E64 in THP-1 macrophages. Results are presented as mean ± SEM, n = 3; * *p*-value < 0.05, ## *p*-value < 0.01, * compared to Act d 1, # compared to control.

Since Act d 1 has been reported to exert pro-inflammatory and pro-allergenic effects in vitro and in vivo only when enzymatically active, we examined its ability to induce IL-1β and IL-6 production in THP-1 cells. To determine whether these effects depend on its protease activity, active and E64-inhibited forms of Act d 1 were used for treatment. The production of both cytokines was significantly reduced in cells treated with the E64-inhibited Act d 1 (Figure 10A,B), further supporting previous findings that Act d 1 protease activity is essential for triggering an immune response.

### 3.6. Impact of Vanilloids on iNOS Expression

**Figure 11:**
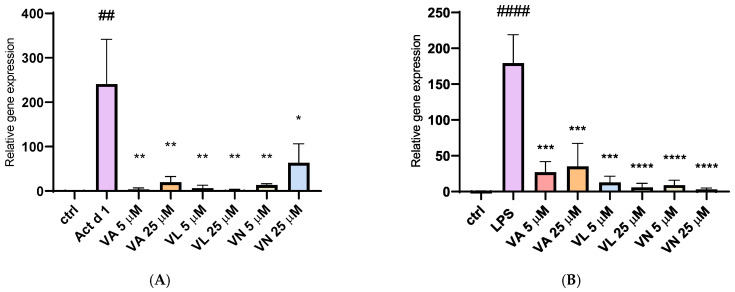
Gene expression of iNOS synthase after stimulation with Act d 1 (**A**) and LPS (**B**) in THP-1 macrophages. Gene expression of iNOS was normalized using expression of housekeeping gene GAPDH and presented as relative gene expression to the control group of untreated cells. Results are presented as mean ± SEM, n = 3; * *p*-value < 0.05, **/## *p*-value < 0.01, *** *p*-value < 0.001, and ####/**** *p*-value < 0.0001, * compared to Act d 1/LPS, # compared to control. VA—vanillyl alcohol, VL—vanillyl laurate, VN—vanillin.

The relative gene expression of inducible nitric oxide synthase (iNOS) was analyzed after isolating total RNA from treated THP-1 cells. Stimulation with both Act d 1 and LPS (0.1 µg/mL) resulted in a significant upregulation of iNOS expression, emphasizing their role in promoting a pro-oxidative environment and M1-like phenotype in THP-1 differentiated macrophages (Figure 11A,B). Pre-treatment with all three tested vanilloids in both concentrations reduced iNOS expression, demonstrating their modulatory potential and suggesting anti-oxidative effects.

### 3.7. Vanilloids Mitigate NF-κB Activation in THP-1 Cells

**Figure 12:**
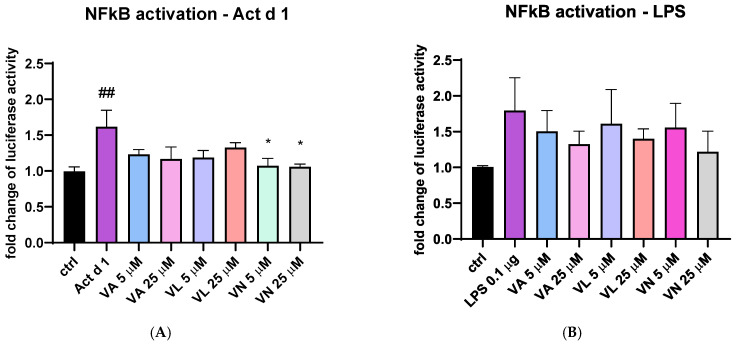
NF-κB activity in THP-1 cells after transfection with NanoLuc^®^ reporter vector with response element. The fold change in luciferase activity was normalized to untreated control and is shown after stimulation with Act d 1 (**A**) and LPS (**B**). Results are presented as mean ± SEM, n = 4; * *p*-value < 0.05, ## *p*-value < 0.01, * compared to Act d 1/LPS, # compared to control. VA—vanillyl alcohol, VL—vanillyl laurate, VN—vanillin.

To additionally explore the effect of vanilloids, THP-1 cells transfected with NanoLuc^®^ reporter vector with NF-κB response element were stimulated with Act d 1 and LPS with or without pre-treatment with the tested vanilloids. Both Act d 1 and LPS induced an increase in luciferase activity compared to the untreated control (Figure 12A and Figure 9B). After stimulation with Act d 1, both concentrations of VN (5 and 25 μM) reduced NF-κB activity to similar levels as the control group (Figure 12A). However, in the LPS stimulated cells, the reduction in NF-κB activity was not decreased with a statistical significance (Figure 12B).

## 4. Discussion

Macrophages are central mediators of the immune response to diverse inflammatory stimuli, including allergens. Their morphological and functional properties, as well as the phenotypic characteristics, are crucial for this function [50]. Our study was designed to analyze the impact of the major kiwifruit allergen Act d 1 on THP-1 macrophages as a model of APC and to test the effects of vanilloids. We used THP-1 macrophages differentiated from monocytes using PMA. Following the changes in receptor expression, we analyzed parameters indicating oxidative stress and NF-κB activation to further characterize the macrophage response to the selected molecules.

Macrophages have crucial and essential role in tissue homeostasis as well as the inflammation process, which has been widely covered by many studies but is still a very enticing research topic. Studies highlight the regulation of macrophage differentiation through the immune response, the interaction with various immune cells such as leukocytes, clearance of apoptotic cells by macrophages and the unique functions and features of macrophages distributed in tissues with diverse environments [21,23,51]. Initial interaction with neighboring cells and sensing the environmental signals is crucial during the initial inflammatory response [23], while their ability to facilitate the resolution and termination of inflammation is crucial in the later phases of inflammation [51]. As a result of all changes they can sense, activation of different inflammatory signaling pathways takes place, inducing expression and secretion of inflammatory cytokines and upregulation of specific receptors, ready to further facilitate the immune response [6,21]. Due to these essential roles in the immunological response, they are widely used in research to explore effects on inflammation and immune modulation [50]. However, obtaining primary human macrophages from blood donors brings certain challenges, such as donor variability and limited cell availability [5,12,50]. Cell lines like THP-1 are widely used to overcome these limitations, as they offer key advantages such as genetic uniformity and reproducibility [12]. Various agents can be used for differentiating THP-1 monocytes into macrophages, including PMA, 1α, 25-dihydroxyvitamin D3 (vD3), a compound that results in macrophages with lower phagocytic abilities and lower secretion of pro-inflammatory cytokines, or macrophage colony stimulating factor (M-CSF)—less used in vitro [12]. Several studies agree that PMA produces the most effective differentiation of THP-1 monocytes to macrophages similar to human PBMCs [12,52,53]. Despite their widespread use, there is no consensus on a standard differentiation protocol of THP-1 using PMA. In this study, in order to use them in further experiments, we optimized the differentiation of THP-1 cells, evaluating the effects of PMA exposure and resting time on cell morphology. A total of nine distinct differentiation conditions were tested, combining varying durations of PMA treatment (24, 48 or 72 h) with different lengths of resting time in PMA-free medium (24, 48 or 72 h). Our findings highlight the critical importance of the resting period following PMA exposure in achieving a complete morphological transition. Notably, cells rested only 24 h post-PMA exposure exhibited no significant increase in cell area, suggesting incomplete differentiation. In contrast, extended resting periods, particularly 72 h, led to substantial increases in cell area, consistent with macrophage morphology. However, while the cell and nuclear areas increased proportionally in most groups, cells exposed to PMA for 72 h displayed a plateau in the nuclear area despite continued cell enlargement. The change in nuclear to cell growth ratio was only significant in the group treated for 72 h with PMA and rested for another 72 h. Since nuclear size in eukaryotic cells is usually correlated with cell size [48], this change may reflect cellular stress due to prolonged culture time. In line with this, analysis of total reduced glutathione (GSH) levels across nine differentiation time points revealed no statistically significant changes; however, a trend toward increased GSH with extended resting in PMA-free medium suggests a gradual restoration of redox balance following PMA exposure, further underlining the importance of resting duration in establishing a stable macrophage cell line. The stability of GSH levels is particularly important in macrophages, where glutathione has been reported to regulate the balance between M1- and M2-like phenotypes, as well as in T cells, where it can influence the dominance of either a Th1 or a Th2 response [54,55]. In our study, recovering to stable GSH levels in untreated cells after differentiation was significant to ensure that polarization did not occur spontaneously, but rather only in response to the applied stimuli. Based on the overall results, we identified 48 h of PMA treatment followed by 72 h of resting as the optimal differentiation protocol, as under these conditions cells exhibited macrophage-like morphology and size and maintained a balanced nuclear-to-cytoplasmic ratio and stable GSH levels not altered by PMA or the differentiation time. These conditions were then used for all downstream experiments.

Macrophage polarization and phenotypic changes are key events in the immune response to various stimuli [50]. Macrophages can polarize into two main phenotypes, M1, pro-inflammatory, and M2, which are associated with anti-inflammatory functions [55]. These two predominant phenotypes very much differ in their function and can be distinguished based on receptor profiles, cytokine secretion and expression of specific genes. M1-like macrophages, characterized by elevated production of ROS, nitrogen species and oxidative enzymes, can lead to activation of transcriptional factors such as NF-κB [55,56]. They typically produce pro-inflammatory cytokines including IL-1β, IL-6 and IFN-γ, and in contrast, M2-like macrophages can lead to production of cytokines such as IL-4, IL-10 and IL-13 [8,57,58]. Studies using established cell lines like THP-1 cells have shown that these polarization states can be achieved in vitro using various stimuli, and the resulting profiles are consistent with in vivo findings. THP-1 cells have been successfully polarized into both M1 and M2 phenotypes using classical stimuli, such as LPS and IL-4, respectively [59]. In addition to classical stimuli with well-characterized polarization effects, studies have investigated the impact of various compounds on macrophage polarization in THP-1 cells. These include exosomes, hyaluronan, glycoproteins, transcriptional regulators, kinase inhibitors and bioactive compounds found in food, electronic cigarettes, etc. [57,60,61,62,63,64]. Such approaches aim to evaluate the potential of these agents to modulate macrophage phenotype and function, contributing to the relevance of a cell culture line such as THP-1 studying changes in macrophage polarization. We used food allergen Act d 1, a cysteine protease, an allergen previously shown to activate NF-κB in epithelial cells and macrophages [17,18], to assess changes in macrophage polarization. LPS was used as a positive control with known pro-inflammatory effect. We analyzed CD80 and CD86, which are typically associated with M1-like pro-inflammatory macrophages phenotype, and CD206 and CD163, associated with M2-like macrophages [6]. Following Act d 1 and LPS stimulations, three different food bioactive compounds, from the family of vanilloids, VA, VL and VN, were used to analyze their impact on the polarization of the stimulated macrophages. Act d 1 and LPS significantly increased CD80, with Act d 1 having even a more pronounced pro-inflammatory shift than LPS, leading to increased receptor expression in over 40% of cells. Other markers tested, including CD86, CD206 and CD163, showed no significant changes, suggesting a limited and specific activation pattern of Act d 1, which acts as a more potent trigger of macrophage activation towards a pro-inflammatory phenotype. We also evaluated whether vanilloids could modulate this response. Both vanillyl laurate (VL) and vanillin (VN) showed a reduction in CD80-positive cell population after Act d 1 stimulation, with only VN having a statistically significant effect. However, CD80 remained above untreated control values, and none of the M2 markers tested were significantly elevated after vanilloid treatment, indicating that while vanilloids have some effect in dampening the inflammatory response, the ability to completely reverse Act d 1-induced polarization is limited. To exclude the possibility that the vanilloids are inhibiting the enzyme Act d 1, thus mitigating the pro-inflammatory effect, we conducted an enzymatic assay and observed no changes in enzymatic activity at different ratios of vanilloids used, meaning that the observed effects are not a result of direct inhibition of the Act d 1 but a cause of the immunomodulatory properties of these molecules.

The shift towards the M1-like pro-inflammatory phenotype that was observed in this study after Act d 1 treatment of PMA-differentiated macrophages is often associated with elevated oxidative stress [6]. In line with this, we analyzed NO and ROS production, as well as catalase activity and GSH levels, and observed a significant increase in ROS, NO and catalase activity after Act d 1 treatment, as well as increases in ROS production and catalase activity after LPS treatment of the PMA-differentiated macrophages. Pre-treatment with VL and VN significantly reduced NO levels after Act d 1 treatment, as well as ROS production to a baseline level. In LPS treated cells, VN leads to a statistically significant decrease in ROS production. Catalase, as one of the key antioxidant enzymes, can be upregulated as a response to increased ROS, as observed in our results. Since not all oxidative stress parameters were equally affected by vanilloid treatment, it appears that these compounds differ in their efficacy depending on the specific oxidative marker assessed or the sensitivity of the analytical method used. This variability may also reflect subtle changes in the measured parameters that fall below the detection thresholds of some of the assays. Reduced glutathione (GSH) was also measured, and while both Act d 1 and LPS led to a decrease, and the vanilloids led to a slight increase, these changes were not statistically significant. Increased GSH concentrations have shown modulatory effects in cultures of antigen-presenting cells such as THP-1, RAW 264.7 and U937, and this effect has been associated with NF-κB and ROS scavenging [65]. However, since in this study we did not analyze the ratio of reduced to oxidized glutathione, there are limitations in commenting analysis of only GSH levels. In general, these results indicate that bioactive food components such as vanilloids, particularly VL and VN, can counteract oxidative stress induced by other stimuli such as food allergens or classic pro-inflammatory agents such as LPS.

As part of the M1-like inflammatory profile, we also analyzed IL-1β and IL-6 secretion, iNOS gene expression and NF-κB pathway activation, all of them being key indicators of inflammation [6,11]. Further supporting the presence of oxidative stress and macrophage polarization, both Act d 1 and LPS significantly upregulated iNOS gene expression, a marker commonly associated with the pro-inflammatory M1 phenotype and a key oxidative mediator [56]. Importantly, pre-treatment with the vanilloids supressed and downregulated iNOS expression, highlighting their immunomodulatory potential and suggesting a shift away from the M1-like macrophage phenotype. Stimulation with Act d 1 as well as with LPS also induced a noticeable increase in pro-inflammatory cytokine secretion, and pre-treatment with VN significantly reduced cytokine levels in all cases tested. However, all of the tested vanilloids reduced IL-6 production after Act d 1 treatment, which was notably less pronounced than in LPS stimulation, suggesting different modulatory potential with stimuli of various strength. Consistent with previous findings, our results show that only enzymatically active Act d 1 statistically induces the production of IL-1β and IL-6, as well as increased catalase activity. Inhibition of its cysteine protease activity with E64 significantly reduced these markers, reinforcing the notion that Act d 1 requires its proteolytic function to elicit an immune response. This supports earlier studies emphasizing the central role of allergen protease activity in modulating innate immune signaling. Using a reporter assay, we also observed an increase in NF-κB activation after both LPS and Act d 1 treatment, and VN successfully inhibited NF-κB activation in Act d 1-stimulated macrophages at both tested concentrations. In the LPS-treated group, although a similar downward trend was observed following VN treatment, the change was not statistically significant. This observation could be attributed to the differences in the strength of the immune response induced by Act d 1 versus LPS. This is best seen in differences in IL-6 production, where Act d 1 induced more than seven times lower secretion of this cytokine compared to LPS. It is a possibility that VN more effectively impacts the less pronounced Act d 1-induced NF-κB activation, compared to the potent activation triggered by a canonical stimulus such as LPS. A key limitation of this study, however, is that these experiments were conducted on a single cell line—THP-1-derrived macrophages. While THP-1 is a cell line that offers important advantages, such as homogenous genetic background and the absence of donor-to donor variability seen in primary cells, it cannot fully recapitulate the complexity of in vivo immune responses. These findings would benefit from future validation in relevant animal models and future studies using donor-derived macrophages and additional immune cell types to fully assess the physiological relevance and translational potential of the observed effects. Future work should aim to investigate the involvement of specific receptors in the activation of signaling cascades, as well as an additional mechanistic insight into the potential immunomodulatory effects.

## 5. Conclusions

The effect of bioactive food components and allergens is not limited to the epithelial barrier and can be significant if these molecules come in contact with immune cells, such as tissue macrophages. In vitro cell lines such as THP-1 can be useful in characterizing these effects. Although it may not be feasible to define a universally standardized THP-1 differentiation protocol for macrophages due to varying experimental conditions across laboratories, key events such as PMA exposure and resting period must be optimized. The phenotype characterization of Act d 1 and LPS treated THP-1 macrophages indicated that Act d 1 is capable of driving partial M1-like polarization in THP-1 macrophages, primarily through the upregulation of CD80 and iNOS expression. The increase in NO, ROS and catalase activity in case of Act d 1 stimulation is consistent with the oxidative environment typically associated with M1-like polarization. Moreover, VN appears to have a modest modulatory affect in alleviating the pro-inflammatory effect of the allergen Act d 1, and both VN and VL expressed anti-oxidative properties as well. Analyzing other markers of NF-κB inflammation, such as NF-κB activation and IL-1β and IL-6 secretion, also showed the promising potential of VN as well as other vanilloids to alter M1-like inflammation in THP-1 macrophages. Together, these findings highlight vanilloids’ ability, with emphasis on vanillin, to reduce key markers of inflammation in THP-1 macrophages through both transcriptional and post-transcriptional mechanisms. While the choice of cell cultures in this study provides valuable insight into the immunomodulatory potential of vanilloids, further validation in animal models and/or human-derived antigen-presenting cells is necessary in the future. Such studies would contribute to a deeper understanding of how dietary or natural compounds influence complex immune responses in even more physiologically relevant systems.

## Figures and Tables

**Table 1:** Human primer sequences and their Tm values.

Gene	Sequence	Gene Accession Number	Tm (°C)
GAPDH	Forward:AGCAATGCCTCCTGCACCACCAAC Reverse:CCGGAGGGGCCATCCACAGTCT	NM_002046.5	65
iNOS	Forward:ACCAGTACGTTTGGCAATGG Reverse:TCAGCATGAAGAGCGATTTCT	AF049656.1	49

**Table 2:** PCR cycle.

Steps	Temperature (°C)	Time	Cycles
Reverse transcription with reverse transcriptase (RT)	42	5 min	1
Inactivation of reverse transcriptase	95	3 min	1
Denaturation	95	10 s	40
Annealing	Tm	20 s	
Elongation	72	20 s	
Final elongation	72	5 min	1

**Table 3:** Combination of different times of incubation in PMA and resting period in the differentiation optimization of THP-1 monocytes into macrophages.

Differentiation	Medium with 100 ng/mL PMA (h)	PMA-Free Medium-Resting Time (h)
1	24	24
2	24	48
3	24	72
4	48	24
5	48	48
6	48	72
7	72	24
8	72	48
9	72	72

## Data Availability

Data is available upon request.
